# Cytoscape stringApp 2.0: Analysis and Visualization of Heterogeneous Biological Networks

**DOI:** 10.1021/acs.jproteome.2c00651

**Published:** 2022-12-13

**Authors:** Nadezhda T. Doncheva, John H. Morris, Henrietta Holze, Rebecca Kirsch, Katerina C. Nastou, Yesid Cuesta-Astroz, Thomas Rattei, Damian Szklarczyk, Christian von Mering, Lars J. Jensen

**Affiliations:** † Novo Nordisk Foundation Center for Protein Research, University of Copenhagen, 2200 Copenhagen, Denmark; ‡ Resource on Biocomputing, Visualization, and Informatics, University of California, San Francisco, California 94143, United States; § Instituto Colombiano de Medicina Tropical, Universidad CES, 055413 Sabaneta, Colombia; ∥ Centre for Microbiology and Environmental Systems Science, University of Vienna, 1030 Vienna, Austria; ⊥ Department of Molecular Life Sciences, University of Zurich, 8057 Zurich, Switzerland; # SIB Swiss Institute of Bioinformatics, 1015 Lausanne, Switzerland

**Keywords:** stringApp, STRING, Cytoscape, omics data, enrichment analysis, heterogeneous networks, cross-species interactions, host−parasite, virus−host

## Abstract

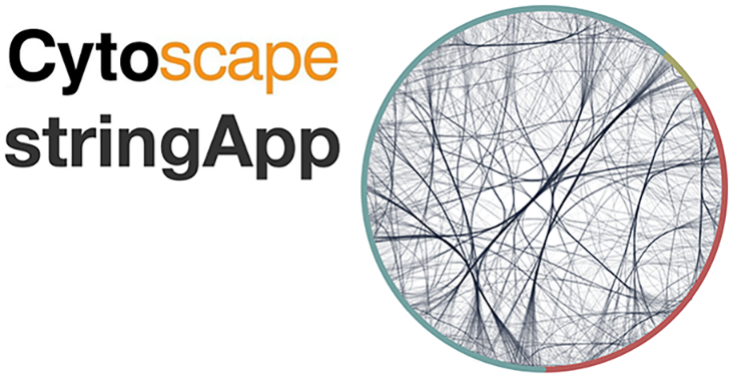

Biological networks are often used to represent complex biological systems, which can contain several types of entities. Analysis and visualization of such networks is supported by the Cytoscape software tool and its many apps. While earlier versions of stringApp focused on providing intraspecies protein–protein interactions from the STRING database, the new stringApp 2.0 greatly improves the support for heterogeneous networks. Here, we highlight new functionality that makes it possible to create networks that contain proteins and interactions from STRING as well as other biological entities and associations from other sources. We exemplify this by complementing a published SARS-CoV-2 interactome with interactions from STRING. We have also extended stringApp with new data and query functionality for protein–protein interactions between eukaryotic parasites and their hosts. We show how this can be used to retrieve and visualize a cross-species network for a malaria parasite, its host, and its vector. Finally, the latest stringApp version has an improved user interface, allows retrieval of both functional associations and physical interactions, and supports group-wise enrichment analysis of different parts of a network to aid biological interpretation. stringApp is freely available at https://apps.cytoscape.org/apps/stringapp.

## Introduction

Networks are a powerful abstraction of biological systems and are useful for modeling and visualizing the complex interplay of the many proteins that make up a cell. Within the field of proteomics, pull-down experiments can be used to produce protein interaction networks, and the results from quantitative proteomics studies are commonly visualized using precomputed networks from databases such as STRING.^1^ Cytoscape^2^ is a widely used platform that is well suited for analysis and visualization of such networks; however, there was previously no straightforward way to import networks from STRING into Cytoscape. The stringApp^3^ solves this by providing easy access to STRING networks within Cytoscape while retaining their distinctive look and much of their functionality.

Whether interpreting the results from omics studies or otherwise trying to understand any biological process or disease, it is important to consider the complex interactions between all the molecular players involved. The STRING database captures this in the form of a functional association network that integrates evidence from multiple sources, including known protein complexes and pathways, automatic text mining of the literature, experimental data, protein coexpression, phylogenetic profiles, and genomic context. The reliability of each association is summarized as a confidence score, which translates to, given the underlying evidence, how sure we can be that two proteins are involved in the same biological process. In addition to the full functional association network, recent versions of STRING provide a physical subnetwork, consisting only of physical protein–protein interactions with confidence scores reflecting the probability of the linked proteins being part of the same protein complex. For convenience, we will in this paper use the word *interactions* to refer to both functional associations and physical interactions.

To make sense of the networks available from databases like STRING, good tools for analysis and visualization are critical. Cytoscape is one such tool. Through its graphical user interface (GUI), users can import protein networks and augment them with data from disparate sources (e.g., proteomics data), analyze them, and visualize protein-centric data on protein networks in many different ways. Cytoscape also has an App Store with hundreds of apps that extend the core functionality with, for example, additional algorithms for network layout and clustering.^4^

One such app is the stringApp, which extends Cytoscape with several types of queries for retrieving networks from STRING, a visual style for the networks similar to that of the STRING web interface, enrichment analysis, and more. The target user group is researchers and scientists, who focus on the biological interpretation of low- and high-throughput data, usually generated by themselves or retrieved from publicly available resources, as well as the generation of different hypotheses based on these data and its analysis.^5−11^ Therefore, in the first stringApp paper, we focused on how the app can be used to visualize data from omics experiments, in particular from quantitative proteomics, on STRING networks.^3^

Here, we present the new stringApp 2.0, focusing on features that are either new or were not covered in the original publication. Several of these focus on making stringApp better suited for working with heterogeneous networks: a new query type that allows retrieval of cross-species networks, functionality to expand networks with interacting proteins from the same or another species, and the ability to convert any Cytoscape network to a STRING-like network, thereby gaining access to most stringApp features. Enrichment analysis has also been greatly improved: functional enrichment can now be automatically retrieved for all groups/clusters of proteins in a network, the results include three times as many categories of terms as previously described, and enriched terms can be added as nodes in the network. The latest stringApp version supports the retrieval of physical interactions as well as functional associations, and it is possible to change a network between the two types. Last but not least, we have made major improvements to the GUI, including a new results panel. We highlight many of the new stringApp features in two use cases that both deal with heterogeneous networks, namely a SARS-CoV-2 interaction map released recently^12^ and a malaria network (including the human host, the parasite *Plasmodium falciparum*, and its vector *Anopheles gambiae*) based on the cross-species interaction data now available in stringApp.

## Methods

### Server-Side Implementation

Cytoscape stringApp retrieves the data from several relational databases, which make it available via web-service application programming interfaces (APIs). The most important is the STRING database,^1^ which contains over 20 billion intraspecies interactions between 67.6 million proteins from 14,094 species. In particular, stringApp uses the STRING API to map identifiers provided by the user to the corresponding STRING protein identifiers and to retrieve functional enrichment.

Besides the protein interactions from STRING, stringApp also provides protein–chemical and chemical–chemical interactions from the STITCH database^13^ as well as cross-species virus–host protein–protein interactions from the Viruses.STRING database.^14^ In addition, stringApp also queries DISEASES^15^ and PubMed to retrieve sets of genes related to a disease or topic of interest, respectively.^3^ Furthermore, each protein or compound is accompanied by additional information, including the subcellular localization data from COMPARTMENTS,^16^ the tissue expression data from TISSUES,^17^ and the drug target classification from Pharos/TCRD^18^ whenever available.

To speed up retrieval of large networks and to support additional types of queries, we store the combined network in a dedicated database, which is accessible via an API. Specifically, we designed this database to support efficient network expansion as well as retrieval of heterogeneous networks, such as cross-species interactions. Two versions of the database exist, one with the full network of functional associations and another with the subnetwork of physical interactions only.

### Host–Parasite Networks

A list of 1,239 host–parasite species pairs was defined based on literature searches, expert knowledge, and public databases (FUNGuild^19^ and GloBI^20^). Only eukaryotic nonplant species already part of STRING were considered as hosts, primarily with a focus on human and other mammals. Parasites were restricted to protozoa, arthropoda, worms, and fungi, and also parasite vectors were included (e.g., *Anopheles* mosquitoes as vectors of malaria parasites that belong to the genus *Plasmodium*).

Functional and physical host–parasite protein pairs were identified using text mining and experimental evidence, largely as described previously for STRING intraspecies pairs.^1^ However, while the confidence scores of STRING functional associations are calculated by benchmarking protein pairs against the KEGG pathway database^21^ (and against Complex Portal^22^ for physical interactions), such a comprehensive gold standard resource is missing for host–parasite protein interactions. To circumvent this issue, we estimated interspecies confidence scores from intraspecies STRING scores,^23^ assuming that how well experimental methods work and how protein relationships are reported in the literature does not differ fundamentally for proteins from the same or different species.

Following the assumption that protein interactions are preserved between orthologous protein pairs,^14,23^ the confidence scores of host–parasite protein pairs were transferred to orthologous proteins in other host–parasite pairs. Orthologous proteins were determined by a reciprocal best hit approach based on Smith–Waterman sequence alignments and self-normalized bit scores obtained from the SIMAP database.^24^ Evidence was transferred within genera for parasites (e.g., *Plasmodium*) and up to class taxonomic level for hosts (e.g., Mammalia), penalizing the scores based on sequence similarity. To obtain the final functional and physical confidence scores for each protein pair, direct and transferred evidence from both experiments and text mining was aggregated as described previously for STRING.^23^ The full pipeline for orthology transfer of host–parasite interactions is available on GitHub (https://github.com/HenriettaHolze/parasite-string-pipeline).

### Client-Side Implementation and Queries

The app itself is implemented in Java using the Cytoscape 3.9 API. The latest version is available on the Cytoscape app store under https://apps.cytoscape.org/apps/stringApp and the source code is publicly available on GitHub https://github.com/RBVI/stringApp under the 2-Clause BSD License.

stringApp 2.0 provides five different queries for creating STRING networks in Cytoscape, and four of them have been previously described.^3^ The *STRING*: *protein query* and *STITCH: compound query* accept as input one or several protein or compound identifiers, map those to STRING/STITCH identifiers, and retrieve the protein–protein or protein–chemical interactions between them from STRING or STITCH, respectively. The *STRING: diseases query* and *STRING: PubMed query* are executed in two steps. First the user enters a disease or topic of interest, for which stringApp retrieves the top-N proteins related to it, and second the app fetches the interactions between these proteins available in the STRING database.

The new type of query in version 2.0 is a *STRING: cross-species query*. As input it takes the scientific names of two species of interest, for which we have at least one cross-species protein–protein interaction with the default confidence score of 0.4. The resulting network consists of the cross-species interactions at the chosen cutoff as well as any intraspecies interactions among the same proteins. stringApp also adds a dedicated visual style with a node color mapping that allows visualization of up to six different species within a network.

For all five query types there is a new option, which allows users to choose the type of network: *full STRING network* and *physical subnetwork*. The confidence cutoff and network type chosen as input are saved in the *Network table*. STRING networks can be converted from one type to the other using the menu *Change confidence or type* or the *Edge* tab in the *STRING Results* panel. Changing the network type causes the stringApp to delete all existing edges and to retrieve new edges of the chosen type for all proteins in the network. Changing the confidence will result in a new network query only if the cutoff is lowered. If the confidence cutoff is increased, stringApp will delete all edges with a confidence lower than the new user-defined one.

Another new feature is that stringApp can convert Cytoscape networks that do not originate from STRING into STRING-like networks using the option *STRINGify network*. The only input parameters needed from the user are a column that contains protein identifiers or names mapped by STRING and the species they belong to. The original edges are lifted over to the mapped nodes, and no additional edges are retrieved from STRING. Technically, this is accomplished by setting the network confidence cutoff to 1.0. This means that edges from STRING can subsequently be added by lowering the cutoff to the default value of 0.4 or another user-specified value. All node and edge attributes from the original network are copied to the new network. By default, unmappable nodes, those without an identifier recognized by STRING for the given species, are kept in the network and their node names are transferred to the new network.

All networks created with stringApp have the *STRING look-and-feel*, which is accomplished by a predefined visual style with a glass ball effect, 3D structure images, and STRING-style labels. To make all information provided to stringApp by the server more accessible to the user, stringApp displays it in a completely new *STRING Results* panel, which contains protein localization filters for the nodes in addition to confidence score filters for the edges.

### stringApp Enrichment

One of the main features of stringApp is the ability to retrieve and visualize functional enrichment for all gene set categories available in the STRING functional enrichment: Gene Ontology annotations,^25^ UniProtKB keywords,^26^ KEGG pathways,^21^ Reactome pathways,^27^ WikiPathways,^28^ Monarch human phenotypes,^29^ Pfam^30^ and SMART^31^ protein domains, InterPro protein features,^32^ and local STRING clusters.^33^ Recently, three new categories were added based on disease-gene associations, subcellular and tissue localization extracted from the DISEASES,^15^ COMPARTMENTS,^16^ and TISSUES^17^ databases, respectively. stringApp uses a RESTful API to send the list of STRING identifiers from the current network or a user-specified selection and receives all enriched terms with a false discovery rate (FDR) < 0.05 from the STRING web server in a JSON file format. The JSON file is parsed to create the enrichment table shown under the *STRING Enrichment* tab in the Cytoscape *Table Panel*. From this table the user can filter the enrichment terms, remove redundant terms, visualize selected enriched terms on the nodes, and export the enrichment results to a file.

STRING also allows for another form of enrichment analysis, namely publication enrichment. For this, each open-access full-text article from PMC and each abstract from PubMed is considered a gene set, consisting of the genes found mentioned in the text by automatic text mining.^33^ This enrichment category is provided separately from the functional enrichment and can be retrieved using the *Enriched publications* menu or button in stringApp. The enriched publications with corresponding title, year of publication and set of genes are displayed in the *STRING Publications* tab in the Cytoscape *Table Panel* and can be sorted by any of the columns, e.g., by year.

The latest version of stringApp is able to retrieve enrichment not only for a single set of nodes but for several nonoverlapping groups of nodes in the network. To define the groups, the user chooses a node attribute column, which could be part of the data imported by the user or, for example, the result of network clustering. Then, stringApp sends a request to the STRING enrichment API for each group, parses the results and displays them as separate enrichment tables in a drop-down menu in the *STRING Enrichment* tab. Each table can be viewed, filtered, and exported independently.

One final new addition to the enrichment functionality is the possibility to add enriched terms as additional nodes to the table. In the *STRING Enrichment* table, the user can right-click an enriched term and add it to the network. The resulting heterogeneous network will contain a new node representing the term (with node type *enriched_term*) and new edges between it and all proteins annotated with it (with interaction type *enrichment*). The information about the enriched term, including the FDR value, the enrichment category, and the number of input and background genes the term annotates, is stored as node attributes.

### Specific Data and Analyses for Use Cases

For the first case study, we use a SARS-CoV-2 interaction network that contains 332 high-confidence protein interactions between SARS-CoV-2 proteins and human proteins.^12^ The interactions were identified by affinity-purification-mass spectrometry (AP-MS) analysis for 26 of the 29 SARS-CoV-2 proteins that could be cloned, tagged and expressed in human cells. The data are publicly available as Supplementary Table 2 of Gordon et al.^12^ and were downloaded on the eighth of July 2022. To make it easier to use the group-wise enrichment functionality of stringApp, we added a column *Bait_ID* that, for each prey, contains the name of the bait (provided as Table S1). All analyses were performed in Cytoscape version 3.9.1 with stringApp version 2.0 and clusterMaker2 app^34^ version 2.2 and are provided as Cytoscape sessions (10.6084/m9.figshare.21313611).

## Results and Discussion

### stringApp at a Glance

The main goal of stringApp remains to provide a seamless connection between two widely used resources, the STRING database and the Cytoscape platform. This is accomplished by (1) facilitating retrieval of STRING interaction networks from within Cytoscape, (2) automatically annotating the nodes and edges with information from STRING and associated databases, (3) providing a visualization that is as close as possible to that of the STRING web resource, and (4) allowing networks created in the STRING web resource to be sent to Cytoscape.

When using stringApp, the first step is to obtain an initial network. This can be done using either of five different query types, or by converting an existing network to a STRING-like network via the new *STRINGify network* functionality. The next step is often to modify the network by increasing/decreasing the confidence score, switching between functional associations and physical interactions, or expanding the network with additional nodes, be it small molecule compounds or additional proteins from the same or another species.

**Figure 1:**
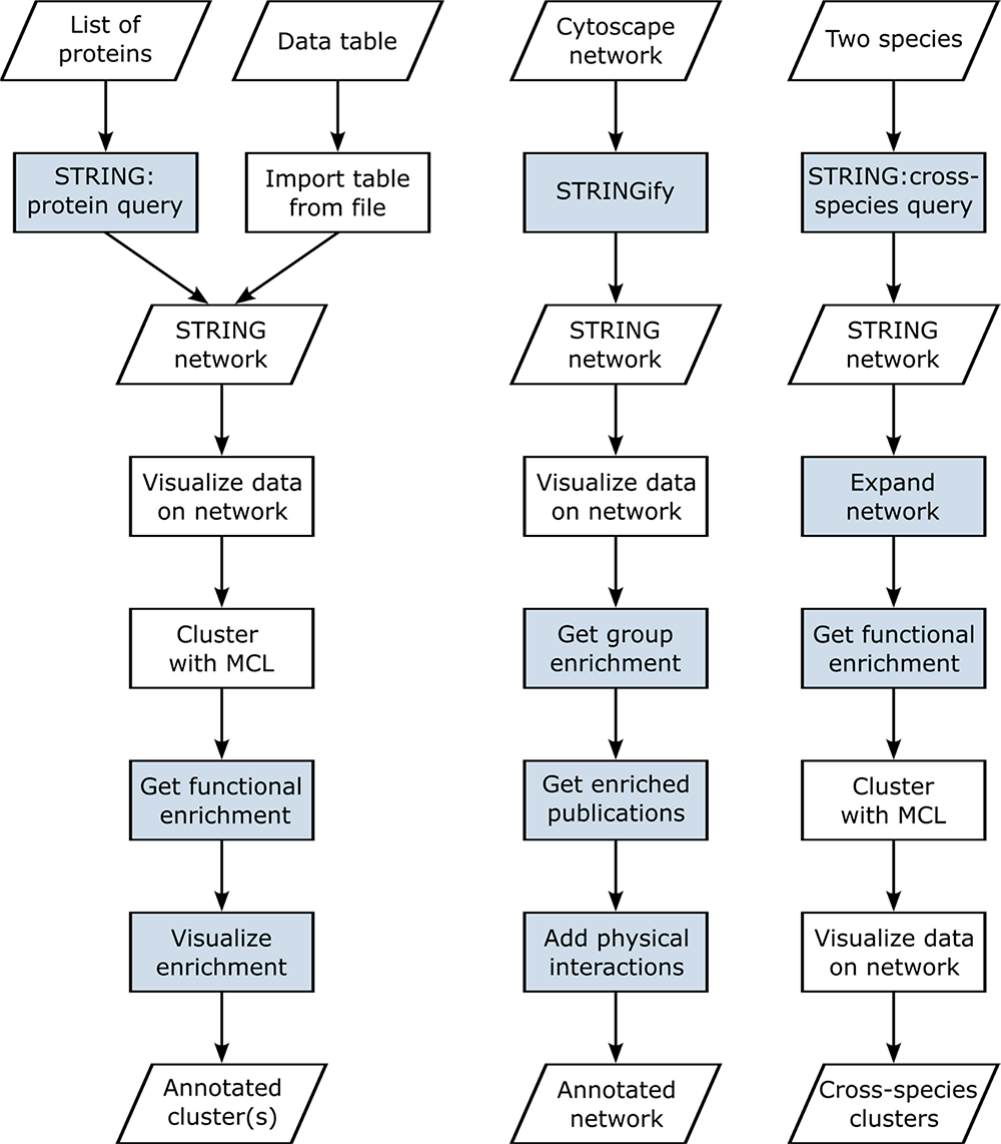
Three example workflows for using stringApp in Cytoscape. While the first one is based on the analysis of proteomics data described in the previous stringApp publication,^3^ the second and third represent the two use cases described here, one focusing on an experimentally determined virus–host network and the other on a host–parasite network retrieved with stringApp. Colored boxes represent functionality implemented by stringApp.

Depending on the biological questions, the next steps can include inspecting individual nodes or edges, visualizing the user’s own data on the network, clustering the network, or performing enrichment analysis. In Figure 1, we show three example workflows, one representing the analysis of proteomics data^35^ shown in the original stringApp publication,^3^ one showing how external data on virus–host interactions can be combined with data from STRING, and finally one highlighting the new cross-species interaction networks in stringApp 2.0. Detailed tutorials with some of the most common workflows can be found here: https://jensenlab.org/training/stringapp/.

### Major New Features

Since the first publication describing stringApp,^3^ we have continued to add new functionality to the app, both to improve it for the existing user base and to make it more broadly useful. Here we will briefly describe the most exciting new features.

**Figure 2:**
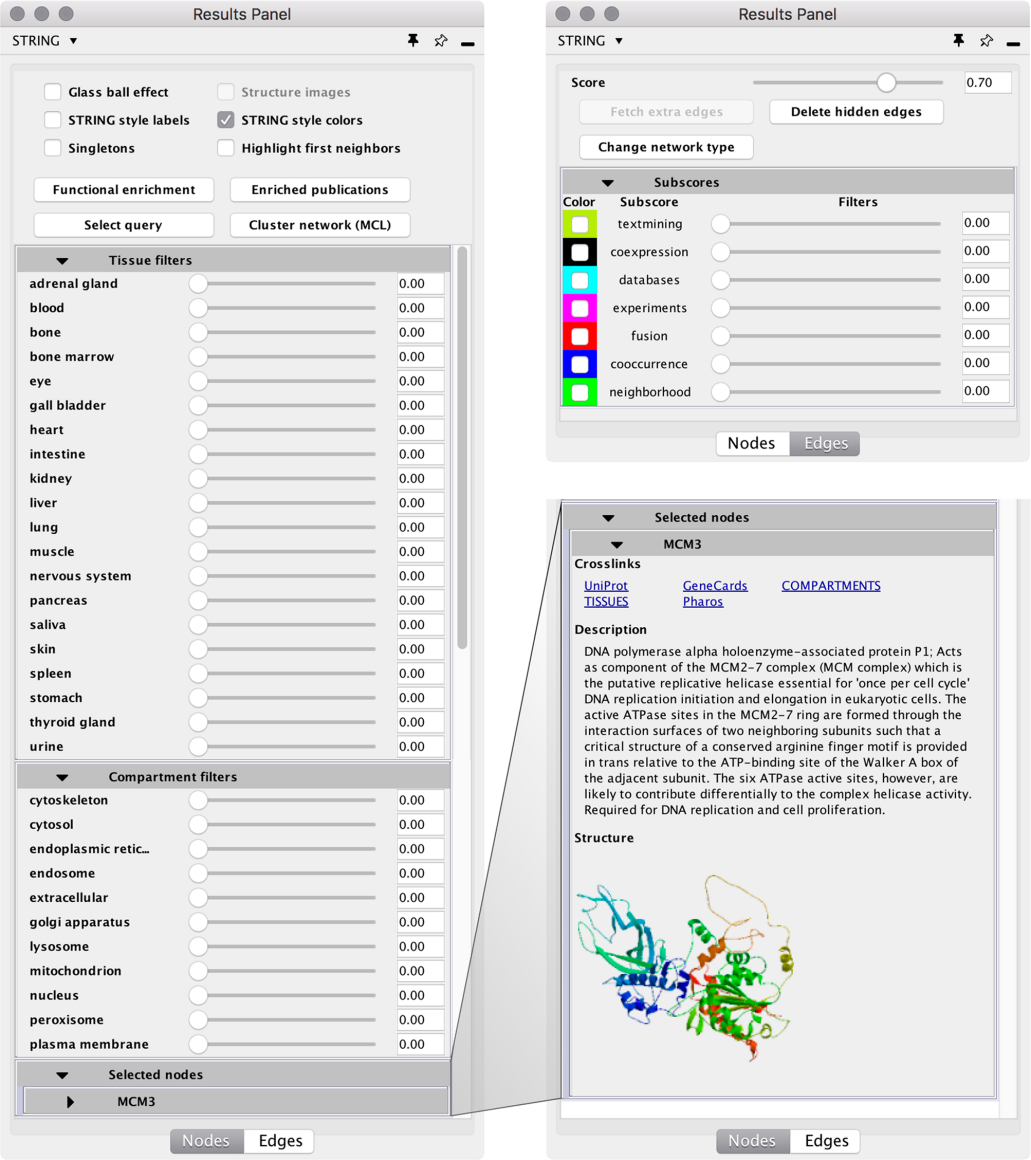
stringApp *Results panel* in Cytoscape. The *Nodes tab* (left) provides quick access to changing different visual properties, running network analysis tasks, and filtering based on tissue and subcellular localization. It also contains a panel with information for each currently selected node, as shown for MCM3 (bottom right). The *Edges tab* allows users to change the network type and confidence as well as to filter and color the edges based on evidence.

We have made many improvements to the GUI, in particular creating a new *Results panel* (Figure 2). This panel provides easy access to much of the information that was previously only accessible through the *Node* and *Edge tables*. It also allows nodes and edges to be filtered based on their subcellular/tissue localization and underlying evidence, respectively. Finally, the *Results panel* provides quick access to many stringApp functions, such as changing the visual properties of the nodes (e.g., the glass ball effect and display of structure images), changing the network type or confidence cutoff, and performing network clustering or enrichment analysis.

We have added the often requested feature of retrieving functional enrichment for each of many groups in a network. This can be particularly useful after clustering a large network, as it allows for functional characterization of the many resulting clusters at once. However, to generalize this functionality, we do not limit it to cluster numbers created by, for example, the clusterMaker2 app,^34^ but allow users to choose any node attribute that defines the groups. The enrichment functionality of stringApp has also been expanded with publication enrichment, which allows users to identify publications that mention surprisingly many of the genes/proteins in their network.

Another common request was to be able to run the enrichment analysis of stringApp on networks from other sources. We have addressed this by implementing new functionality, called *STRINGify*, which allows users to convert any network in Cytoscape to a new network that contains the same set of nodes and edges, but is recognized by the stringApp. This action gives users access to all the additional node attributes retrieved by stringApp for the nodes that can be mapped to STRING as well as most of the stringApp features, including enrichment analysis. It also allows users to easily combine their own interaction networks with information from STRING.

Starting with version 11.5, STRING distinguishes between two types of interactions, namely functional associations and physical interactions. stringApp provides the user with the options to choose which type of network they want when querying and to change the type of an existing network. Both types of networks come with confidence scores, which indicate how likely two proteins are to participate in the same pathway, in the case of functional associations, or be part of the same protein complex, in the case of physical interactions.

While not part of STRING, the backend database for stringApp has been expanded with more than 58 million functional associations and half a million physical interactions for host–parasite pairs. To provide better access to these, as well as to the existing virus–host interactions, we have implemented a dedicated query interface for cross-species interactions. This new *STRING: cross-species query* takes two different species as input and retrieves a network with interactions within and between the selected species. Currently, we have interaction data for 92 parasites and 124 hosts as well as for 239 viruses and their 319 hosts.

We exemplify the new features of stringApp through two case studies that both focus on heterogeneous networks. In the first we show how a published SARS-CoV-2 interaction map can be augmented and analyzed with stringApp, and in the second how a cross-species network for malaria can be retrieved and visualized. An example of how stringApp can be used to create and visualize networks of chemical compounds, proteins, and enriched terms can be found in the first stringApp publication^3^ and in the recent paper about the Arena3D^web^ interactive web tool.^36^

### SARS-CoV-2 Interaction Map

A new functionality in stringApp is the ability to take a user-provided interaction network and map the proteins to STRING. This augments the mapped proteins with all the additional data normally provided by stringApp, such as protein localization data, allows STRING interactions to subsequently be added to the network, and enables the enrichment functionality.

To exemplify this, we start by importing an affinity purification-based interaction network for SARS-CoV-2 proteins and human host proteins into Cytoscape. We downloaded the supplementary table with the SARS-CoV-2 interaction map from Gordon et al.,^12^ in which the viral proteins were used as baits, and their human interaction partners were identified as preys. We used the standard *Import network from file* functionality to load this interaction network into Cytoscape. Next, we used the new *STRINGify network* functionality of stringApp to create a new network, in which all the human proteins have been mapped to the corresponding proteins in STRING. In this new network, the viral proteins are left as-is, and the original edges are copied over. Although this network is not a true STRING network per se, as neither the viral proteins nor any of the edges are from STRING, this mapping is sufficient to enable key features such as the enrichment analysis functionality of stringApp for the network.

The latest version of stringApp can automatically run enrichment analysis individually for each cluster or group of proteins in a network. In this use case, we define the groups based on the SARS-CoV-2 bait proteins, so that the prey proteins for each bait are considered a separate group. The result is an enrichment panel, which contains a separate table for each bait protein, listing the statistically significantly enriched terms among its prey proteins. Using this functionality on the SARS-CoV-2 interaction map reveals, among other things, that the viral glycoprotein orf8 interacts mainly with host glycoproteins, that the membrane protein orf9c interacts exclusively with host membrane proteins, and that the RNA-binding protein nsp8 interacts with RNA-binding host proteins. More interestingly, it also shows that the nsp9 protein, which attenuates nuclear transport,^37^ interacts with host proteins involved in nuclear pore disassembly, and that five interactors of orf10 are involved in Cul2-RING ubiquitin ligase hijacking by SARS-CoV-2. This illustrates how the group-wise enrichment analysis in stringApp can be a powerful tool for interpreting protein interaction data from, for example, AP-MS experiments.

**Figure 3:**
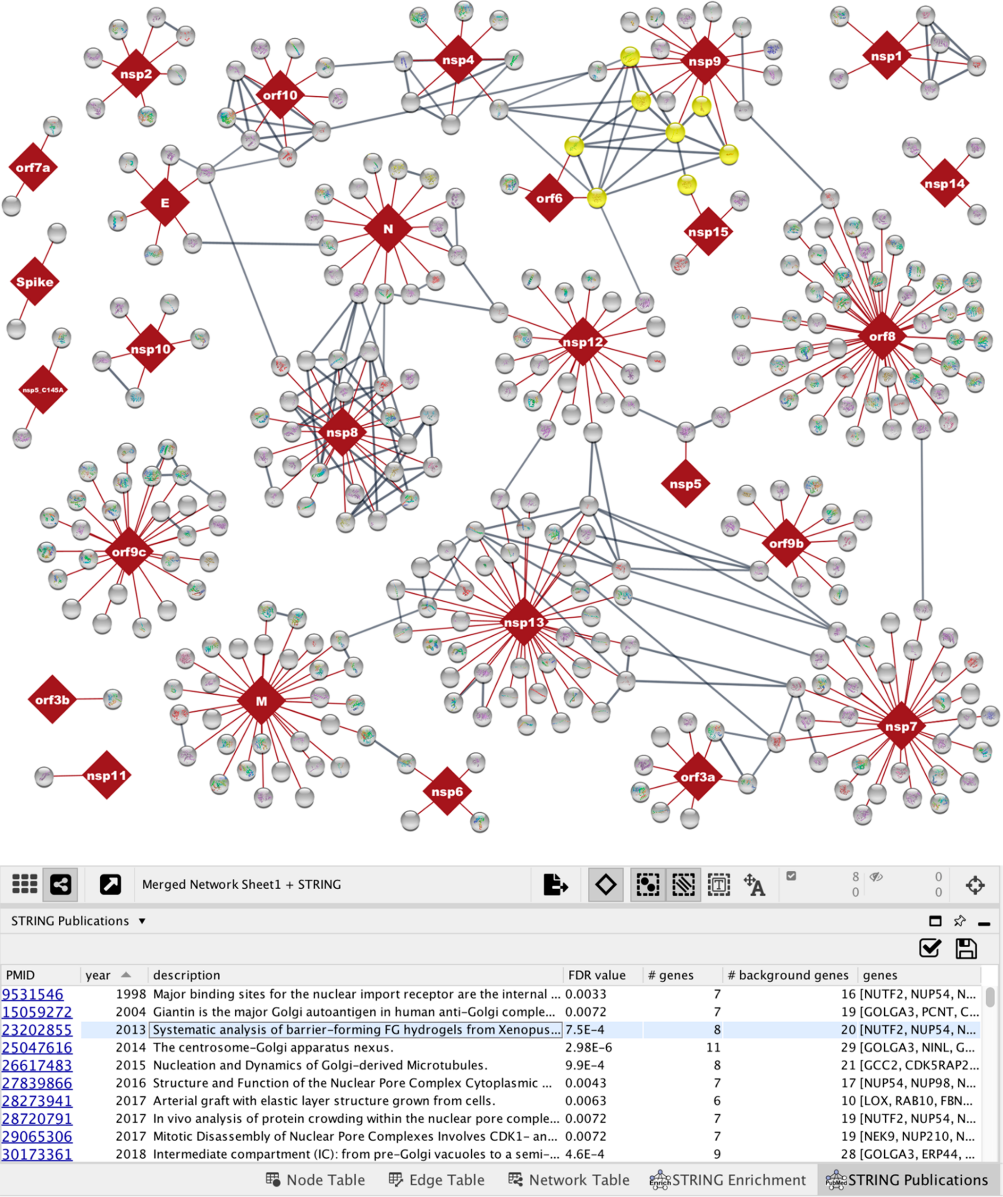
Visualization of a STRINGified virus–host network. The network contains 332 high-confidence protein–protein interactions (red edges) between SARS-CoV-2 (dark red nodes) and human proteins (gray nodes) identified by affinity-purification mass spectrometry.^12^ stringApp was used to fetch high-confidence physical interactions between the human proteins (score cutoff ≥0.8, gray edges) and to retrieve enriched publications. One of the publications and the proteins it mentions are highlighted in the *STRING Publications table* (light blue line) and in the network view (yellow colored nodes).

Another new type of enrichment analysis added to stringApp is publication enrichment, in which each publication (PubMed abstract or full-text open access article) is considered as a gene set, namely the genes identified in it by automatic text mining.^33^ Network-wide publication enrichment analysis of the SARS-CoV-2 interaction map identified a paper about barrier-forming FG hydrogels^38^ (Figure 3), which have been implicated in how viruses challenge the selectivity of host nuclear pores.^39^ The proteins mentioned in the enriched paper (Figure 3, yellow nodes) interact with several SARS-CoV-2 proteins (nsp9, nsp15, and orf6).

Finally, we check if the host proteins associated with the aforementioned paper interact with each other, despite them not interacting with the same viral bait proteins. To do so, we change the network type to *physical* and lower the confidence cutoff from 1.0 (the default for a network without STRING edges) to 0.8, causing stringApp to retrieve high-confidence physical protein interactions from STRING. The host protein interactions retrieved in this way show that the proteins found in the publication enrichment analysis indeed interact with each other (Figure 3).

### Malaria Cross-Species Network

In the second use case, we highlight some of the new stringApp features related to cross-species networks between hosts and parasites, including network expansion with interaction partners from other species. We also showcase how the visualization of STRING networks can be improved using built-in Cytoscape functionality and other Cytoscape apps, such as clusterMaker2.^34^ For this, we chose to look at malaria, a mosquito-borne parasitic infectious disease that affects humans.^40^

**Figure 4:**
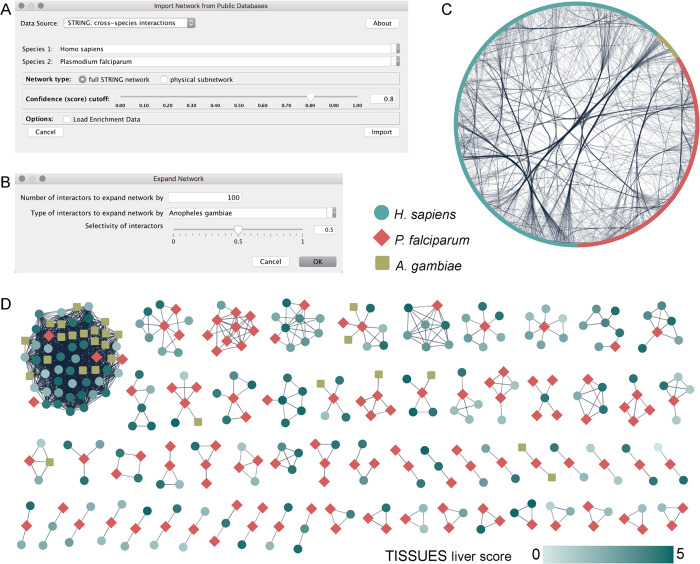
Analysis of a malaria cross-species network with stringApp in Cytoscape. (A) stringApp user interface for cross-species queries. (B) stringApp *Expand network* options dialogue. (C) Network of *H. sapiens* (teal), *P. falciparum* (red), and *A. gambiae* (lime) proteins and their high-confidence inter- and intraspecies interactions (score cutoff 0.8). (D) All cross-species clusters after applying Markov clustering (MCL) on the network. Human proteins are colored based on the TISSUES confidence scores for liver.

We start by creating a STRING network of functional associations using the new *STRING: cross-species query* with the two species *Homo sapiens* and *Plasmodium falciparum* (Figure 4A). If we used the default confidence cutoff of 0.4 for this pair of species, we would get a huge network of several thousands of nodes and hundreds of thousands of edges. To avoid this, the confidence cutoff should be increased in the query options (for example to 0.8) *before* starting the network retrieval. The resulting high-confidence network contains 847 nodes and 3,904 edges, of which 306 nodes represent *P. falciparum* proteins and 1,001 edges are cross-species interactions. By default, stringApp assigns the color teal to the host proteins and the color red to the parasite proteins (first and second species in the query, respectively).

The next step is to add proteins from *Anopheles gambiae*, the major malaria vector,^41^ that spreads the *P. falciparum* protozoan through its bite. As we are interested in which mosquito proteins interact with the parasite, we first select all *P. falciparum* proteins using the built-in Cytoscape selection filter with the node attribute *stringdb::species*. We then use the *Expand network* functionality of stringApp to add up to 100 proteins from *A. gambiae*, which interact with the selected proteins with a confidence of at least 0.8 (Figure 4B). In this case, the network is expanded by 35 mosquito proteins and 357 inter- and intraspecies interactions, which by default are placed in a grid layout next to the network. To show the overall interaction pattern between the three species, we apply the built-in *Attribute Circle layout* (with *name* as the attribute) and use the Cytoscape functionality to bundle all edges (Figure 4C). However, even at this high confidence cutoff, the network is very dense and not so easy to explore.

The functional enrichment functionality of stringApp can help provide more insight about the proteins in the network. We can retrieve it for the human proteins by pressing the *Functional enrichment* button in the *Results panel* and choosing *H. sapiens* from the species list. The analysis reveals several enriched tissues, in particular *liver*, which is in accordance with the known tissue tropism of *P. falciparum*.^42,43^

A common way to simplify such large and dense networks is to perform network clustering, which will identify groups of tightly connected nodes, in this case, proteins likely to be in the same biological process or pathway based on their functional associations. For user convenience, there is a *Cluster network* (*MCL*) button in the stringApp *Results panel*, which internally calls the clusterMaker2 app.^34^ This way, the clustering is already configured to use the provided STRING edge confidence scores and create a view of the clustered network. The dialogue suggests a default inflation value of 4, which is usually suitable for dense STRING networks. In the resulting clustered network, we identify several clusters that contain proteins from all three species, including examples of human and mosquito proteins that interact with the same parasite protein (Figure 4D). To highlight which of the interacting human proteins are expressed in liver tissue, we use the tissue expression information retrieved by stringApp from the TISSUES database together with a continuous node color mapping, while bypassing the mapping to set the node color of the other two species.

## Conclusions

In summary, we have extended the Cytoscape stringApp with several new features and substantially improved its functionality. In particular, we designed a new user interface for exploring the available information, enabled automated enrichment analysis for several groups of nodes at the same time, added a new query type for retrieving cross-species networks as well as the option to STRINGify networks not created with stringApp. Many of these extensions focus on the support of heterogeneous networks, such as networks that contain nodes/edges from both STRING and another source, networks with proteins from several different species, and networks of proteins and selected terms from enrichment analysis. We present most of the new functionality in two use cases that both involve a cross-species network for human pathogens. The improvements in stringApp 2.0 also lay the foundation for providing other types of cross-species networks, such as symbiosis and microbiomes, once such data become available.

## Data Availability

stringApp is freely available at https://apps.cytoscape.org/apps/stringapp.

## Supporting Information Available

Table S1: SARS-CoV-2-human interaction pairs extracted from Supplementary Table 2 of Gordon et al., *Nature*, 2020 (XLSX)

## References

[ref1] Szklarczyk D.; Gable A. L.; Nastou K. C.; Lyon D.; Kirsch R.; Pyysalo S.; Doncheva N. T.; Legeay M.; Fang T.; Bork P.; Jensen L. J.; von Mering C. The STRING Database in 2021: Customizable Protein-Protein Networks, and Functional Characterization of User-Uploaded Gene/measurement Sets. Nucleic Acids Res. 2021, 49 (D1), D605–D612. 10.1093/nar/gkaa1074. 33237311 PMC7779004

[ref2] Shannon P.; Markiel A.; Ozier O.; Baliga N. S.; Wang J. T.; Ramage D.; Amin N.; Schwikowski B.; Ideker T. Cytoscape: A Software Environment for Integrated Models of Biomolecular Interaction Networks. Genome Res. 2003, 13 (11), 2498–2504. 10.1101/gr.1239303. 14597658 PMC403769

[ref3] Doncheva N. T.; Morris J. H.; Gorodkin J.; Jensen L. J. Cytoscape StringApp: Network Analysis and Visualization of Proteomics Data. J. Proteome Res. 2019, 18 (2), 623–632. 10.1021/acs.jproteome.8b00702. 30450911 PMC6800166

[ref4] Lotia S.; Montojo J.; Dong Y.; Bader G. D.; Pico A. R. Cytoscape App Store. Bioinformatics 2013, 29 (10), 1350–1351. 10.1093/bioinformatics/btt138. 23595664 PMC3654709

[ref5] Drexler H. C. A.; Vockel M.; Polaschegg C.; Frye M.; Peters K.; Vestweber D. Vascular Endothelial Receptor Tyrosine Phosphatase: Identification of Novel Substrates Related to Junctions and a Ternary Complex with EPHB4 and TIE2. Mol. Cell. Proteomics 2019, 18 (10), 2058–2077. 10.1074/mcp.RA119.001716. 31427368 PMC6773558

[ref6] Kim Y. S.; Fan R.; Lith S. C.; Dicke A.-K.; Drexler H. C. A.; Kremer L.; Kuempel-Rink N.; Hekking L.; Stehling M.; Bedzhov I. Rap1 Controls Epiblast Morphogenesis in Sync with the Pluripotency States Transition. Dev. Cell 2022, 57 (16), 1937–1956. 10.1016/j.devcel.2022.07.011. 35998584

[ref7] Nielsen J. E.; Honoré B.; Vestergård K.; Maltesen R. G.; Christiansen G.; Bøge A. U.; Kristensen S. R.; Pedersen S. Shotgun-Based Proteomics of Extracellular Vesicles in Alzheimer’s Disease Reveals Biomarkers Involved in Immunological and Coagulation Pathways. Sci. Rep. 2021, 11 (1), 18518 10.1038/s41598-021-97969-y. 34531462 PMC8445922

[ref8] Kieffer F.; Pronot M.; Gay A.-S.; Debayle D.; Gwizdek C. Proteomics Datasets of Developing Rat Brain: Synaptic Proteome and SUMO2/3-Ylome. Data Brief 2022, 42, 108151 10.1016/j.dib.2022.108151. 35516005 PMC9062222

[ref9] Hallal M.; Braga-Lagache S.; Jankovic J.; Simillion C.; Bruggmann R.; Uldry A.-C.; Allam R.; Heller M.; Bonadies N. Inference of Kinase-Signaling Networks in Human Myeloid Cell Line Models by Phosphoproteomics Using Kinase Activity Enrichment Analysis (KAEA). BMC Cancer 2021, 21 (1), 789 10.1186/s12885-021-08479-z. 34238254 PMC8268341

[ref10] Versace E.; Sgadò P.; George J.; Loveland J. L.; Ward J.; Thorpe P.; Jensen L. J.; Spencer K. A.; Paracchini S.; Vallortigara G. Light-Induced Asymmetries in Embryonic Retinal Gene Expression Are Mediated by the Vascular System and Extracellular Matrix. Sci. Rep. 2022, 12 (1), 12086 10.1038/s41598-022-14963-8. 35840576 PMC9287303

[ref11] Lee E.-H.; Park J.-Y.; Kwon H.-J.; Han P.-L. Repeated Exposure with Short-Term Behavioral Stress Resolves Pre-Existing Stress-Induced Depressive-like Behavior in Mice. Nat. Commun. 2021, 12 (1), 6682 10.1038/s41467-021-26968-4. 34795225 PMC8602389

[ref12] Gordon D. E.; Jang G. M.; Bouhaddou M.; Xu J.; Obernier K.; White K. M.; O’Meara M. J.; Rezelj V. V.; Guo J. Z.; Swaney D. L.; Tummino T. A.; Hüttenhain R.; Kaake R. M.; Richards A. L.; Tutuncuoglu B.; Foussard H.; Batra J.; Haas K.; Modak M.; Kim M.; Haas P.; Polacco B. J.; Braberg H.; Fabius J. M.; Eckhardt M.; Soucheray M.; Bennett M. J.; Cakir M.; McGregor M. J.; Li Q.; Meyer B.; Roesch F.; Vallet T.; Mac Kain A.; Miorin L.; Moreno E.; Naing Z. Z. C.; Zhou Y.; Peng S.; Shi Y.; Zhang Z.; Shen W.; Kirby I. T.; Melnyk J. E.; Chorba J. S.; Lou K.; Dai S. A.; Barrio-Hernandez I.; Memon D.; Hernandez-Armenta C.; Lyu J.; Mathy C. J. P.; Perica T.; Pilla K. B.; Ganesan S. J.; Saltzberg D. J.; Rakesh R.; Liu X.; Rosenthal S. B.; Calviello L.; Venkataramanan S.; Liboy-Lugo J.; Lin Y.; Huang X.-P.; Liu Y.; Wankowicz S. A.; Bohn M.; Safari M.; Ugur F. S.; Koh C.; Savar N. S.; Tran Q. D.; Shengjuler D.; Fletcher S. J.; O’Neal M. C.; Cai Y.; Chang J. C. J.; Broadhurst D. J.; Klippsten S.; Sharp P. P.; Wenzell N. A.; Kuzuoglu-Ozturk D.; Wang H.-Y.; Trenker R.; Young J. M.; Cavero D. A.; Hiatt J.; Roth T. L.; Rathore U.; Subramanian A.; Noack J.; Hubert M.; Stroud R. M.; Frankel A. D.; Rosenberg O. S.; Verba K. A.; Agard D. A.; Ott M.; Emerman M.; Jura N.; von Zastrow M.; Verdin E.; Ashworth A.; Schwartz O.; d’Enfert C.; Mukherjee S.; Jacobson M.; Malik H. S.; Fujimori D. G.; Ideker T.; Craik C. S.; Floor S. N.; Fraser J. S.; Gross J. D.; Sali A.; Roth B. L.; Ruggero D.; Taunton J.; Kortemme T.; Beltrao P.; Vignuzzi M.; García-Sastre A.; Shokat K. M.; Shoichet B. K.; Krogan N. J. A SARS-CoV-2 Protein Interaction Map Reveals Targets for Drug Repurposing. Nature 2020, 583 (7816), 459–468. 10.1038/s41586-020-2286-9. 32353859 PMC7431030

[ref13] Szklarczyk D.; Santos A.; von Mering C.; Jensen L. J.; Bork P.; Kuhn M. STITCH 5: Augmenting Protein-Chemical Interaction Networks with Tissue and Affinity Data. Nucleic Acids Res. 2016, 44 (D1), D380–D384. 10.1093/nar/gkv1277. 26590256 PMC4702904

[ref14] Cook H. V.; Doncheva N. T.; Szklarczyk D.; von Mering C.; Jensen L. J. Viruses.STRING: A Virus-Host Protein-Protein Interaction Database. Viruses 2018, 10 (10), 519 10.3390/v10100519. 30249048 PMC6213343

[ref15] Grissa D.; Junge A.; Oprea T. I.; Jensen L. J. Diseases 2.0: A Weekly Updated Database of Disease-Gene Associations from Text Mining and Data Integration. Database 2022, 2022, baac019 10.1093/database/baac019. 35348648 PMC9216524

[ref16] Binder J. X.; Pletscher-Frankild S.; Tsafou K.; Stolte C.; O’Donoghue S. I.; Schneider R.; Jensen L. J. COMPARTMENTS: Unification and Visualization of Protein Subcellular Localization Evidence. Database 2014, 2014, bau012 10.1093/database/bau012. 24573882 PMC3935310

[ref17] Palasca O.; Santos A.; Stolte C.; Gorodkin J.; Jensen L. J. TISSUES 2.0: An Integrative Web Resource on Mammalian Tissue Expression. Database 2018, 2018, bay003 10.1093/database/bay003. 29617745 PMC5808782

[ref18] Sheils T. K.; Mathias S. L.; Kelleher K. J.; Siramshetty V. B.; Nguyen D.-T.; Bologa C. G.; Jensen L. J.; Vidović D.; Koleti A.; Schürer S. C.; Waller A.; Yang J. J.; Holmes J.; Bocci G.; Southall N.; Dharkar P.; Mathé E.; Simeonov A.; Oprea T. I. TCRD and Pharos 2021: Mining the Human Proteome for Disease Biology. Nucleic Acids Res. 2021, 49 (D1), D1334–D1346. 10.1093/nar/gkaa993. 33156327 PMC7778974

[ref19] Nguyen N. H.; Song Z.; Bates S. T.; Branco S.; Tedersoo L.; Menke J.; Schilling J. S.; Kennedy P. G. FUNGuild: An Open Annotation Tool for Parsing Fungal Community Datasets by Ecological Guild. Fungal Ecol. 2016, 20, 241–248. 10.1016/j.funeco.2015.06.006.

[ref20] Poelen J. H.; Simons J. D.; Mungall C. J. Global Biotic Interactions: An Open Infrastructure to Share and Analyze Species-Interaction Datasets. Ecol. Inform. 2014, 24, 148–159. 10.1016/j.ecoinf.2014.08.005.

[ref21] Kanehisa M.; Sato Y.; Kawashima M.; Furumichi M.; Tanabe M. KEGG as a Reference Resource for Gene and Protein Annotation. Nucleic Acids Res. 2016, 44 (D1), D457–D462. 10.1093/nar/gkv1070. 26476454 PMC4702792

[ref22] Meldal B. H. M.; Bye-A-Jee H.; Gajdoš L.; Hammerová Z.; Horácková A.; Melicher F.; Perfetto L.; Pokorný D.; Lopez M. R.; Türková A.; Wong E. D.; Xie Z.; Casanova E. B.; Del-Toro N.; Koch M.; Porras P.; Hermjakob H.; Orchard S. Complex Portal 2018: Extended Content and Enhanced Visualization Tools for Macromolecular Complexes. Nucleic Acids Res. 2019, 47 (D1), D550–D558. 10.1093/nar/gky1001. 30357405 PMC6323931

[ref23] von Mering C.; Jensen L. J.; Snel B.; Hooper S. D.; Krupp M.; Foglierini M.; Jouffre N.; Huynen M. A.; Bork P. STRING: Known and Predicted Protein-Protein Associations, Integrated and Transferred across Organisms. Nucleic Acids Res. 2004, 33 (Database issue), D433–D437. 10.1093/nar/gki005. PMC539959 15608232

[ref24] Arnold R.; Goldenberg F.; Mewes H.-W.; Rattei T. SIMAP--the Database of All-against-All Protein Sequence Similarities and Annotations with New Interfaces and Increased Coverage. Nucleic Acids Res. 2014, 42 (Database issue), D279–D284. 10.1093/nar/gkt970. 24165881 PMC3965014

[ref25] The Gene Ontology Resource: 20 Years and Still GOing Strong. Nucleic Acids Res. 2019, 47 (D1), D330–D338. 10.1093/nar/gky1055. 30395331 PMC6323945

[ref26] UniProt: The Universal Protein Knowledgebase in 2021. Nucleic Acids Res. 2021, 49 (D1), D480–D489. 10.1093/nar/gkaa1100. 33237286 PMC7778908

[ref27] Jassal B.; Matthews L.; Viteri G.; Gong C.; Lorente P.; Fabregat A.; Sidiropoulos K.; Cook J.; Gillespie M.; Haw R.; Loney F.; May B.; Milacic M.; Rothfels K.; Sevilla C.; Shamovsky V.; Shorser S.; Varusai T.; Weiser J.; Wu G.; Stein L.; Hermjakob H.; D’Eustachio P. The Reactome Pathway Knowledgebase. Nucleic Acids Res. 2019, 48 (D1), D498–D503. 10.1093/nar/gkz1031. PMC7145712 31691815

[ref28] Martens M.; Ammar A.; Riutta A.; Waagmeester A.; Slenter D. N.; Hanspers K.; A Miller R.; Digles D.; Lopes E. N.; Ehrhart F.; Dupuis L. J.; Winckers L. A.; Coort S. L.; Willighagen E. L.; Evelo C. T.; Pico A. R.; Kutmon M. WikiPathways: Connecting Communities. Nucleic Acids Res. 2021, 49 (D1), D613–D621. 10.1093/nar/gkaa1024. 33211851 PMC7779061

[ref29] Shefchek K. A.; Harris N. L.; Gargano M.; Matentzoglu N.; Unni D.; Brush M.; Keith D.; Conlin T.; Vasilevsky N.; Zhang X. A.; Balhoff J. P.; Babb L.; Bello S. M.; Blau H.; Bradford Y.; Carbon S.; Carmody L.; Chan L. E.; Cipriani V.; Cuzick A.; Della Rocca M.; Dunn N.; Essaid S.; Fey P.; Grove C.; Gourdine J.-P.; Hamosh A.; Harris M.; Helbig I.; Hoatlin M.; Joachimiak M.; Jupp S.; Lett K. B.; Lewis S. E.; McNamara C.; Pendlington Z. M.; Pilgrim C.; Putman T.; Ravanmehr V.; Reese J.; Riggs E.; Robb S.; Roncaglia P.; Seager J.; Segerdell E.; Similuk M.; Storm A. L.; Thaxon C.; Thessen A.; Jacobsen J. O. B.; McMurry J. A.; Groza T.; Köhler S.; Smedley D.; Robinson P. N.; Mungall C. J.; Haendel M. A.; Munoz-Torres M. C.; Osumi-Sutherland D. The Monarch Initiative in 2019: An Integrative Data and Analytic Platform Connecting Phenotypes to Genotypes across Species. Nucleic Acids Res. 2020, 48 (D1), D704–D715. 10.1093/nar/gkz997. 31701156 PMC7056945

[ref30] Mistry J.; Chuguransky S.; Williams L.; Qureshi M.; Salazar G. A.; Sonnhammer E. L. L.; Tosatto S. C. E.; Paladin L.; Raj S.; Richardson L. J.; Finn R. D.; Bateman A. Pfam: The Protein Families Database in 2021. Nucleic Acids Res. 2021, 49 (D1), D412–D419. 10.1093/nar/gkaa913. 33125078 PMC7779014

[ref31] Letunic I.; Bork P. 20 Years of the SMART Protein Domain Annotation Resource. Nucleic Acids Res. 2018, 46 (D1), D493–D496. 10.1093/nar/gkx922. 29040681 PMC5753352

[ref32] Blum M.; Chang H.-Y.; Chuguransky S.; Grego T.; Kandasaamy S.; Mitchell A.; Nuka G.; Paysan-Lafosse T.; Qureshi M.; Raj S.; Richardson L.; Salazar G. A.; Williams L.; Bork P.; Bridge A.; Gough J.; Haft D. H.; Letunic I.; Marchler-Bauer A.; Mi H.; Natale D. A.; Necci M.; Orengo C. A.; Pandurangan A. P.; Rivoire C.; Sigrist C. J. A.; Sillitoe I.; Thanki N.; Thomas P. D.; Tosatto S. C. E.; Wu C. H.; Bateman A.; Finn R. D. The InterPro Protein Families and Domains Database: 20 Years on. Nucleic Acids Res. 2021, 49 (D1), D344–D354. 10.1093/nar/gkaa977. 33156333 PMC7778928

[ref33] Szklarczyk D.; Gable A. L.; Lyon D.; Junge A.; Wyder S.; Huerta-Cepas J.; Simonovic M.; Doncheva N. T.; Morris J. H.; Bork P.; Jensen L. J.; von Mering C. STRING v11: Protein-Protein Association Networks with Increased Coverage, Supporting Functional Discovery in Genome-Wide Experimental Datasets. Nucleic Acids Res. 2019, 47 (D1), D607–D613. 10.1093/nar/gky1131. 30476243 PMC6323986

[ref34] Morris J. H.; Apeltsin L.; Newman A. M.; Baumbach J.; Wittkop T.; Su G.; Bader G. D.; Ferrin T. E. clusterMaker: A Multi-Algorithm Clustering Plugin for Cytoscape. BMC Bioinformatics 2011, 12, 436 10.1186/1471-2105-12-436. 22070249 PMC3262844

[ref35] Francavilla C.; Lupia M.; Tsafou K.; Villa A.; Kowalczyk K.; Rakownikow Jersie-Christensen R.; Bertalot G.; Confalonieri S.; Brunak S.; Jensen L. J.; Cavallaro U.; Olsen J. V. Phosphoproteomics of Primary Cells Reveals Druggable Kinase Signatures in Ovarian Cancer. Cell Rep. 2017, 18 (13), 3242–3256. 10.1016/j.celrep.2017.03.015. 28355574 PMC5382236

[ref36] Kokoli M.; Karatzas E.; Baltoumas F. A.; Schneider R.; Pafilis E.; Paragkamian S.; Doncheva N. T.; Jensen L. J.; Pavlopoulos G. A. Arena3D^web^: Interactive 3D Visualization of Multilayered Networks Supporting Multiple Directional Information Channels, Clustering Analysis and Application Integration. bioRxiv 2022, 10.1101/2022.10.01.510435. PMC10227371 37260509

[ref37] Makiyama K.; Hazawa M.; Kobayashi A.; Lim K.; Voon D. C.; Wong R. W. NSP9 of SARS-CoV-2 Attenuates Nuclear Transport by Hampering Nucleoporin 62 Dynamics and Functions in Host Cells. Biochem. Biophys. Res. Commun. 2022, 586, 137–142. 10.1016/j.bbrc.2021.11.046. 34844119 PMC8604569

[ref38] Labokha A. A.; Gradmann S.; Frey S.; Hülsmann B. B.; Urlaub H.; Baldus M.; Görlich D. Systematic Analysis of Barrier-Forming FG Hydrogels from Xenopus Nuclear Pore Complexes. EMBO J. 2012, 32 (2), 204–218. 10.1038/emboj.2012.302. 23202855 PMC3553378

[ref39] Labokha A. A.; Fassati A. Viruses Challenge Selectivity Barrier of Nuclear Pores. Viruses 2013, 5 (10), 2410–2423. 10.3390/v5102410. 24084236 PMC3814595

[ref40] Walter K.; John C. C. Malaria. JAMA 2022, 327 (6), 597 10.1001/jama.2021.21468. 35133414

[ref41] Sinka M. E.; Bangs M. J.; Manguin S.; Rubio-Palis Y.; Chareonviriyaphap T.; Coetzee M.; Mbogo C. M.; Hemingway J.; Patil A. P.; Temperley W. H.; Gething P. W.; Kabaria C. W.; Burkot T. R.; Harbach R. E.; Hay S. I. A Global Map of Dominant Malaria Vectors. Parasit. Vectors 2012, 5, 69 10.1186/1756-3305-5-69. 22475528 PMC3349467

[ref42] Mirsky I. A.; von Brecht R.; Williams L. D. Hepatic Dysfunction In Malaria. Science 1944, 99 (2558), 20–21. 10.1126/science.99.2558.20. 17844554

[ref43] Jain A.; Kaushik R.; Kaushik R. M. Malarial Hepatopathy: Clinical Profile and Association with Other Malarial Complications. Acta Trop. 2016, 159, 95–105. 10.1016/j.actatropica.2016.03.031. 27019056

